# Diagnosis and Management of a Triple Infection with *Leptospira* spp., Hepatitis A Virus, and Epstein–Barr Virus: A Rare Occurrence with High Hepatotoxic Effect

**DOI:** 10.3390/healthcare11040597

**Published:** 2023-02-17

**Authors:** Norberth-Istvan Varga, Diana-Maria Mateescu, Rodica Anamaria Negrean, Florin George Horhat, Iulia-Cristina Bagiu, Shiva Charana Kodimala, Satya Sai Sri Bandi, Razvan Mihai Horhat, Delia Ioana Horhat, Ion Cristian Mot, Bogdan Miutescu

**Affiliations:** 1 Department of Infectious Diseases, Victor Babes University of Medicine and Pharmacy Timisoara, E. Murgu Square, Nr. 2, 300041 Timisoara, Romania; 2 Faculty of Medicine and Pharmacy, University of Oradea, 410073 Oradea, Romania; 3 Multidisciplinary Research Center on Antimicrobial Resistance (MULTI-REZ), Microbiology Department, “Victor Babes” University of Medicine and Pharmacy, 300041 Timisoara, Romania; 4 MediCiti Institute of Medical Sciences, NTR University of Health Sciences, Hyderabad 501401, India; 5 Malla Reddy Institute of Medical Sciences, Suraram Main Road 138, Hyderabad 500055, India; 6 Department of Conservative Dentistry and Endodontics, Faculty of Dental Medicine, “Victor Babes” University of Medicine and Pharmacy Timisoara, Eftimie Murgu Square 2, 300041 Timisoara, Romania; 7 Ear-Nose-Throat Department, “Victor Babes” University of Medicine and Pharmacy Timisoara, 2 Eftimie Murgu Sq, 300041 Timisoara, Romania; 8 Department of Gastroenterology and Hepatology, “Victor Babes” University of Medicine and Pharmacy Timisoara, Eftimie Murgu Square 2, 300041 Timisoara, Romania

**Keywords:** hepatitis A virus, Epstein–Barr virus, leptospirosis, hepatic cytolysis, acute hepatitis

## Abstract

The etiology of acute hepatic cytolysis is complex, and a thorough laboratory investigation is needed to find the causative agent and guide the clinician toward a specific treatment. Viral hepatitis A is a well-known cause of acute hepatitis, but other viruses and bacteria can lead to or contribute to liver damage. We report the case of a young male patient with triple infection with hepatitis A virus, Epstein–Barr virus, and *Leptospira* spp. To our knowledge, this is the first case of an HAV, EBV, and Leptospira triple infection, and it aims to bring awareness about the possibility of double or triple infection with such pathogens that are highly cytotoxic for the liver tissue since all three pathogens are known to cause or contribute to the onset of acute hepatitis. It was deduced that the source of the infection likely happened during a two-week visit to the countryside in Romania, returning 16 days before the onset of symptoms. The evolution was favorable receiving treatment with amoxicillin/clavulanic acid (1200 mg/8 h); glucose 5% 500 mL/day; 0.9% saline 500 mL/day; phenobarbital 1 tablet/day (200 mg); vitamins B1 and B6 and a complex of vitamin C and D3 and zinc. Lactulose syrup was also administered when the patient had no bowel movement for more than 24 h to prevent the onset of hepatic encephalopathy, and the patient was discharged after 20 days. This case suggests that a detailed anamnesis can raise suspicion about more uncommon causes of hepatic cytolysis and lead to a broader and more complex laboratory investigation, thus improving the quality of patient care. Yet, this is the only case previously reported to compare different management options and patient outcomes.

## 1. Introduction

An infection with the hepatitis A virus (HAV) is one of the most prevalent causes of acute viral hepatitis across the globe. Despite many decades’ worth of study, the pathogenic processes behind hepatitis A are still only partially known. As the replication of HAV is not cytopathic in vitro, a commonly held belief has been that virus-specific cytotoxic T lymphocytes cause liver damage [1]. On the other hand, there is growing information that natural killer (NK) cells, non-specific CD8+ T cells, and even CD8+ T cells that are particular to HAV may all participate in liver injury during HAV infection [2]. In addition, host factors’ genetic structure differences, such as T cell immunoglobulin-1 and IL-18 binding protein, are associated with the severity of hepatitis A, varying from mild hepatitis to fulminant hepatitis in patients with apparently similar features [3].

Besides HAV, other viruses are known to cause liver injury. Epstein–Barr virus (EBV) causes infectious mononucleosis in younger people, including children and young adults [4]. It can induce a mild and transient rise in transaminases, but in very uncommon instances, it may trigger serious liver damage and possibly acute liver failure (ALF). Liver transplantation is the only therapy effective in curing EBV-associated ALF [5]. However, only about 0.20% of adult cases of acute liver failure are related to EBV, making it an extremely uncommon form of the disease. Young individuals under the age of 40 are more likely to have symptoms of infectious mononucleosis, which is consistent with the fact that this age group is more likely to be infected with the virus. Previous findings have shown that patients with intact immunity can also develop ALF, which indicates that other unidentified health issues may also be implicated in the progression of EBV-associated liver failure. In adults 60 years or older, there were only two incidents of EBV-associated ALF in the literature; nevertheless, both had deficient immunity [6].

Lastly, another pathogen that triggers liver cytotoxicity, with endemicity in tropical regions but also identified in Romania, is the *Leptospira* spp. The spirochete known as Leptospira is responsible for the infection known as leptospirosis, a zoonosis that has spread all over the globe, although it is most common in tropical regions [7]. It is spread by a wide number of different animals, including cows and rats, which become infected either through direct contact with these animals or their urine or indirectly by consuming or coming into contact with water that has been polluted by the urine of such animals [8]. The clinical manifestations are heterogeneous, with most individuals exhibiting either no or mild symptoms. Unusually, leptospirosis manifests itself in two phases, starting with fever, myalgias, liver damage, and bleeding marking the first phase. In contrast, the second stage is characterized by jaundice resulting from hepatic insufficiency [9]. Weil’s illness is a form of severe leptospirosis characterized by hepatic failure with jaundice and abrupt renal failure, linked with significant fatality rates [10]. The diagnosis was arrived at using serological methods and DNA detection using PCR. The treatment consists of providing basic life support as well as administering antibiotics.

The current study aims to report a rare case of triple concomitant infection with hepatitis A virus, Epstein–Barr virus, and Leptospira in a young male patient who presented with severe symptoms of acute hepatitis. All three infections are known to cause or contribute to the onset of acute hepatitis directly. After a thorough anamnesis, we concluded that the patient may have contracted all three infections while spending two weeks in the countryside. Our report aims to bring awareness about the possibility of double or triple infections with liver-damaging pathogenic agents. To our knowledge, this is the first case of an HAV, EBV, and Leptospira triple infection.

## 2. Case Presentation

A 23-year-old male with no other known medical conditions presented at the Victor Babeș Hospital of Infectious Diseases with an influenced clinical status, with myalgia, headaches, drowsiness, nausea, asthenia, loss of appetite, and high fever (>38.5 °C), cervical adenopathy and jaundice. According to the patient, the signs and symptoms appeared five days prior, and jaundice appeared two days prior. He was clinically and biologically evaluated, and his liver enzymes showed a severe hepatic cytolysis syndrome, with elevated levels of alanine-aminotransferase (3911 U/L), aspartate aminotransferase (2384 U/L), and total bilirubin (4.9 mg/dL). Abdominal ultrasonography was insignificant. Urinalysis revealed hematuria and the presence of bilirubin and urobilinogen in urine.

During a detailed history taking, we discovered that the patient had spent two weeks on holiday in the countryside, approximately 16 days before the onset of symptoms. According to the patient, during his holiday, he bathed in a local river, ate unwashed fruits and vegetables bought or received from locals, and attended a wedding where he drank a large amount of alcohol. He was unaware of any other chronic diseases and took no chronic medication. He states that his urine is darker and that he has not had a bowel movement for six days.

Physical examination revealed jaundice of the skin and the eyes, mild bilateral cervical adenopathy with palpable but small-sized, non-tender lymph nodes, no palpable axillary and inguinal lymph nodes, normal pharynx, uvula, and tonsils, with no signs of acute pharyngitis, normal body temperature, normal cardiac function with a blood pressure of 113/73 mmHg, a heart rate of 73 bpm, oxygen saturation of 100% on ambient air, normal vesicular murmur on both lung fields, no abdominal pain at palpation and no clinical signs of meningitis.

He was admitted and several laboratory tests were performed to find the causative agent. Serological testing revealed positive IgM anti-HAV (hepatitis A virus) antibodies, positive IgM anti-VCA-EBV (Epstein–Barr) antibodies, and a positive complement fixation reaction for Leptospira. HBs antigens and anti-HCV antibodies were negative. The results for the laryngeal and nasal exudate, along with the urine culture tests, were also negative. A protein electrophoresis test was performed, with normal total protein levels (6.79 g/dL), hypoalbuminemia (42.05%; normal range between 55.8–65.0%), and hypergammaglobulinemia (26.4%; normal range between 11.5–8.6%).

After the positive lab tests, the patient started receiving intravenous (IV) amoxicillin/clavulanic acid (1200 mg/8 h); IV glucose 5% 500 mL/day; IV 0.9% saline 500 mL/day; phenobarbital 1 tablet/day (200 mg); vitamins B1 and B6 and a complex of vitamin C and D3 and zinc. Lactulose syrup was also administered when the patient had no stools for more than 24 h to prevent the onset of hepatic encephalopathy. In order to monitor the patient’s liver and kidney functions, several sets of blood and urine tests and a CT scan have been performed, as presented in Table 1.

**Figure 1:**
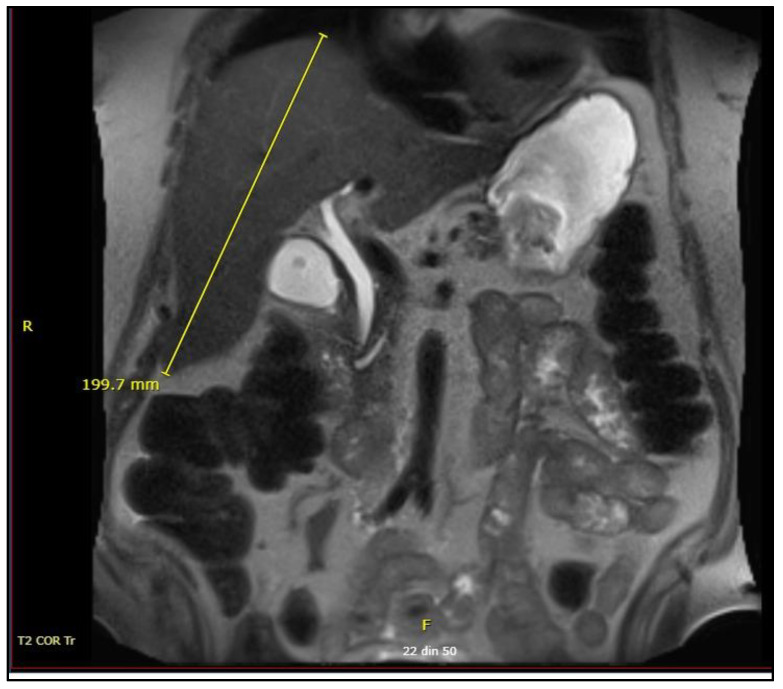
Computed tomography (CT) of the abdomen; R—Right; F—Frontal view.

During the first days after admission, a physical examination revealed that the patient still presented jaundice and bilateral cervical adenopathy with enlarged, non-tender lymph nodes; at superficial abdominal palpation, the patient did not accuse pain, but the inferior edge of the liver was palpable 2–3 cm below the costal margin. We decided to perform a CT scan of the patient’s abdomen, which revealed hepatomegaly but no intrahepatic or extrahepatic biliary obstruction, and normal-sized spleen and kidneys, as seen in Figure 1.

We performed another set of serological tests three days after admission to confirm the previous findings. Again, both IgM anti-HAV antibodies and IgM anti-VCA-EBV antibodies were positive. The treatment continued until the 12th day after admission. Body temperature remained within normal limits during treatment, and gingival bleeding, myalgia, headaches, fatigue, and loss of appetite ceded. No intestinal transit disorders have been observed. The patient did not accuse pain at abdominal palpation. The inferior edge of the liver was palpable at approximately 2–3 cm below the costal margin, with a slow but progressive improvement. Jaundice and hyperchromic urine persisted.

On the 12th day after admission, antibiotic treatment was changed from IV amoxicillin/clavulanic acid (1200 mg/8 h) to IV ceftriaxone (2 g/12 h), administered for seven days. During this treatment, the overall clinical state of the patient improved considerably. On physical examination, the cervical lymph nodes and the inferior margin of the liver were no longer palpable. Jaundice and urine color also improved. After the antibiotic therapy was completed, another complement fixation test for Leptospira was performed, with negative results.

The patient was discharged after 20 days of hospitalization with great improvement in clinical status, no palpable cervical lymph nodes, normal body temperature, normal appetite, no abdominal pain, no palpable inferior margin of the liver, normal urine color, and normal cardiac and pulmonary function. Nevertheless, it would have been interesting to observe the evolution and laboratory analysis even after discharge, although it was no longer justified to keep the patient admitted and consume valuable hospital resources.

## 3. Discussion

### 3.1. Case Features

**Figure 2:**
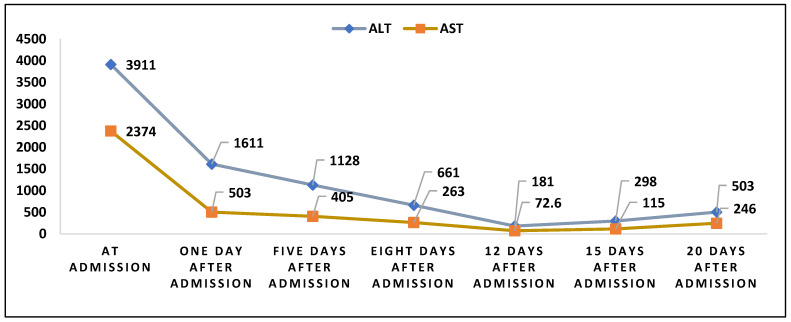
The evolution of ALT and AST levels during hospitalization.

Liver transaminases are measured in evaluating acute viral hepatitis when their levels grow more than tenfold their normal range. ALT is more specific for hepatic cytolysis because it is found only in the liver, whereas AST is also in the heart and skeletal muscle [11]. In acute hepatitis A, mild cholestasis syndrome may be present, but the hepatic cytolysis syndrome is more severe [12]. Interestingly, our patient showed elevated levels of ALT and AST at admission, probably because infectious mononucleosis and leptospirosis can cause a certain degree of liver injury. Peak levels were present at admission, followed by a steep decline during treatment, as seen in Figure 2.

Liver damage causes decreased protein synthesis, leading to an elevated prothrombin time (PT), with a higher risk of bleeding [13]. A decrease of 50% in the prothrombin concentration means a severe form of hepatitis, and a decrease below 30% means fulminant hepatitis. Our patient had normal PT levels at admission but slightly increased during the first two weeks of hospitalization, with the highest level eight days after admission.

The evolution of acute hepatitis A usually involves an incubation period ranging from 15 to 45 days, followed by a prodromal or pre-icteric phase, in which non-specific symptoms may occur [14]. Our patient presented with asthenia, drowsiness, nausea, myalgia, headaches, and loss of appetite. The phase before jaundice onset lasts up to two weeks, followed by the icteric phase, which may last for several weeks. Our patient reports that jaundice appeared only three days after the onset of symptoms. The coinfection might have caused the early appearance of jaundice with EBV or Leptospira. Hepatitis A is more characterized by a hepatic cytolysis syndrome than a cholestatic syndrome. Usually, the level of total bilirubin in acute hepatitis A is mildly or moderately elevated.

**Figure 3:**
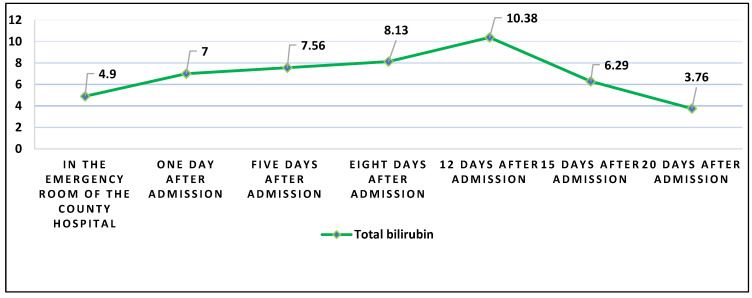
The evolution of total bilirubin levels during hospitalization.

On the other hand, EBV rarely leads to mild jaundice, and on the 12th day of hospitalization, the Leptospira infection was already under antibiotic treatment. This led us to conclude that his peak level of total bilirubin (10.38 mg/dL) on the 12th day of treatment is caused by the severe destruction of liver cells that took place in the early period of disease and is caused mainly by the hepatitis A virus with a synergic effect of the coinfection with Leptospira and EBV. His bilirubin levels did not decrease, but on the contrary, they continued to increase until the end of the second week of hospitalization, reaching a peak of 10.38 mg/dL, as described in Figure 3.

Considering the patient presented to the hospital five days after the onset of symptoms and taking the incubation period into account, we concluded that he probably came into contact with the hepatitis A virus during his vacation in the countryside. Our speculation is also based on the fact that he ate unwashed fruits and vegetables that he received or bought from local growers. The timeframe of the hepatitis A virus infection also probably coincides with the infection with the Epstein–Barr virus.

Infectious mononucleosis, colloquially known as “the kissing disease,” is an infectious, self-limiting disease caused by the Epstein–Barr virus. The source of infection is exclusively human, and it can spread from a person with an acute symptomatic or asymptomatic infection or in recovery. The incubation period can last between four to seven weeks, and the onset of symptoms varies with age. For young adults, signs, and symptoms typically include fever, which can be as high as 39 °C and last for up to two weeks, sore throat with exudative, hypertrophic, and hyperemic pharyngitis accompanied by dysphagia, and swollen lateral cervical lymph nodes [15].

**Figure 4:**
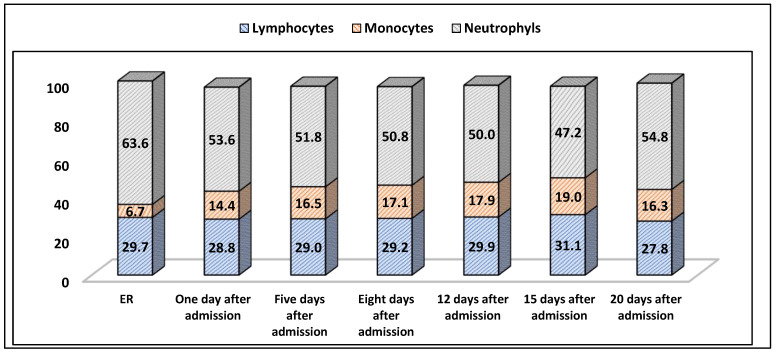
The patient’s white blood cell count in evolution.

The Epstein–Barr virus can also cause mild liver damage, sometimes hepatomegaly, with elevated levels of AST and ALT. Serologically, IgM anti-VCA-EBV antibodies appear early in the EBV infection and have been proven to disappear within four to six weeks. IgG antibodies, however, persist for the rest of the patient’s life. An important laboratory finding is a leukocytosis with elevated lymphocyte and monocyte levels (above 50%), with a peak in the second or third week after the onset of symptoms [16]. Lymphocyte and monocyte levels peaked on the 15th day after admission, growing over 50% of total leukocytes, as seen in Figure 4.

Considering that our patients’ levels of lymphocytes and monocytes peaked around the end of the second week after hospital admission, it is possible that the infectious mononucleosis was in the second or early third week of evolution when the blood tests were drawn. We noted that he did not present any signs of acute pharyngitis at admission, but he did test positive for IgM anti-VCA-EBV antibodies on the second day. Therefore, we concluded that his disease may have started just a few days before admission to our clinic, probably in the same period as the onset of jaundice and acute hepatitis A symptoms. This implies that he encountered the Epstein–Barr virus approximately a month before admission, which coincides with the period he states he started his holiday in the countryside.

Leptospirosis is an infectious disease caused by bacteria of the genus Leptospira. The source of infection is represented by animal carriers such as rodents, cats, dogs, horses, pigs, and so on [17]. The animal host eliminates live bacteria through urine, contaminating the soil and water bodies such as ponds and irrigation canals. Transmission can be made through direct contact with the infected animal, but it is more often through contact with contaminated water. Risk factors for leptospirosis are more often related to recreational practices and hobbies that include contact with water. Still, professional activities can expose someone to infection, such as veterinarians and farmers [18]. The bacteria can enter the body through skin injuries, but it is believed to penetrate intact skin. It then enters the bloodstream, causing the first phase of the disease called leptospiremia, followed by dissemination through various organs, such as the liver, the lungs, or the kidneys, causing different signs and symptoms that vary depending on the affected organ. The size of the inoculum influences the duration of incubation and the severity of the disease. After the acute phase of the disease, or leptospiremia, when the bacteria can be found in the blood, opsonizing antibodies are detected around ten days after infection, and the leptospires disappear from the blood. The antibodies are responsible for eliminating the bacteria and the inflammatory reactions that produce secondary lesions in the damaged organs. Leptospira persists in the renal tubules for several weeks after being eliminated from the blood.

Laboratory confirmation of leptospirosis is needed to certify the diagnosis, where the microscopic agglutination test (MAT) is the gold standard in certifying the diagnosis. It uses live antigens from different serogroups of Leptospira, and it gives a positive result when the antibodies from the infected blood sample come into contact with the specific antigens. The agglutination reaction is examined in darkfield microscopy [19]. Considering that IgM anti-Leptospira antibodies appear in the blood approximately ten days after infection, the MAT can be falsely negative if the test is performed sooner. We performed the complement fixation test (CFT), another antibody detection test that can be used in certifying the leptospirosis diagnosis. Our results were positive only the first time, the second day after admission, and then antibiotic therapy was administered. The second time we performed the complement fixation test after antibiotic treatment, the results were negative.

After an incubation period that can range between two days and three weeks, the onset of symptoms is usually brutal, with fever and severely altered clinical status. Two clinical forms have been described according to the degree of renal damage. The most common form is anicteric leptospirosis, characterized by flu-like symptoms: high fever, headaches, myalgia, loss of appetite, rarely a non-pruritic skin rash, conjunctivitis, and abdominal pain. This form is self-limited, and the general clinical state improves without antibiotics in five or six days. On the other hand, icteric leptospirosis, also known as Weil’s disease, is more dangerous because it usually implies various degrees of multi-visceral damage, such as liver damage (hepatic manifestations include jaundice, hepatic cytolysis with elevated transaminases), kidney damage (renal manifestations include nephritis with acute renal insufficiency, proteinuria, or even oliguria), heart damage (myocarditis, pericarditis), and lung damage (chest pain, coughing, and hemoptysis) [20].

Our patient did not present multi-visceral manifestations, and his serum creatinine levels were within the normal range during hospitalization. No cardiac, pulmonary, or ocular damage has been observed. The leptospirosis was in the early period of evolution when he was admitted to our clinic, and the antibiotic treatment started. Leptospira is known to be sensitive to amoxicillin and received IV amoxicillin/clavulanic acid (1200 mg/8 h). It is possible that he developed the more common, anicteric form of leptospirosis, and his jaundice was not caused by the bacteria but by the two viruses—HAV and EBV.

Considering that the onset of symptoms coincided for all three infections and that the patient bathed in a local river two weeks before that, we believe that he contracted all three infections while spending his two-week vacation in the countryside. Nevertheless, most likely, HAV was the dominant infection considering the clinical features of the patient. EBV infection comes with mild elevation of liver tests, whereas the most common form of leptospirosis is the anicteric type without jaundice. Moreover, the leptospira complement fixation test was negative 12 days after admission.

### 3.2. Similar Findings

Other cases that were previously reported identified more severe hepatotoxicity after Leptospira infection in young adults in their 20s, even though there was no EBV or HAV involvement [21]. A 23-year-old male was taken to the emergency room with a 5-day record of fever, myalgia, jaundice, a rash, hematemesis, and two days of anuria. No medical, surgical, or contributing clinical records were present. The respective patient was not an alcoholic, smoker, or drug user like our patient, and there was no travel history. However, the patient often fished in rivers. The AST and ALT were close to normal values at admission, but the total bilirubin was extremely elevated at 28 mg/dL, the INR at 2.7, serum creatinine at 6 mg/dL, and the serum urea at 106 mg/dL. Following a CT scan of the chest and abdomen revealing widespread micronodular microvascular syndrome, antibiotic treatment with ceftriaxone and spiramycin was initiated.

The first patient suffered a severe feverish sickness accompanied by jaundice, myalgia, acute renal damage, and considerable gastrointestinal bleeding, most likely caused partly by a median arcuate ligament syndrome. In Weil’s illness, serum bilirubin is often considerably increased, accompanied by modest elevations of transaminases and alkaline phosphatase [22]. In this instance, serum transaminases were initially modestly elevated, accompanied by a rise in total bilirubin that was higher than 60 mg/dL. After arterial embolization and cephalic duodeno-pancreatectomy, transaminase levels in the blood started to rise. The median arcuate ligament syndrome undoubtedly had a significant part in the gastrointestinal bleeding and associated problems that ultimately led to the patient’s death. Numerous collateral arteries formed to adjust for the decreased celiac artery blood circulation. Leptospirosis-related vasculitis probably triggered the bleeding in such extremely vulnerable auxiliary arteries.

In the second case described by the literature, the early manifestations were less common, as the patient had abrupt neurological symptoms and an altered degree of awareness. Due to the severity of the severe liver failure, a liver transplant was necessary. In total, 10–15% of individuals [23] are affected by neuro-leptospirosis, and the most prevalent neurological symptom is aseptic meningoencephalitis [24]. Dysfunction of the cerebellum has also been documented and is believed to impact fewer than 5% of patients [25]. Neurologic involvement in leptospirosis is caused by capillary endothelial injury and vasculitis, with a typically favorable outcome. More interesting is that the patient had ciprofloxacin prophylaxis, which is often helpful against leptospirosis infections.

Infections with leptospirosis often cause liver damage, which may manifest as mild abnormalities in blood liver function tests or severe hepatic failure. Previously reported clinical manifestations of liver illness in leptospirosis include hyperplasia of Kupffer cells, cholestasis, cell infiltration in portal regions, and sometimes severe necrosis of hepatocytes leading to hepatic failure [26]. After liver transplantation, significant hepatocyte necrosis was found on histological examination of the resected liver in these two instances. As evidenced by the instances reported below, liver damage may be sudden, severe, and permanent, occasionally resulting in a patient fatality.

Therefore, diagnosing the patients as early as possible is crucial to prevent fatal complications. The most frequent test used to detect leptospirosis is serology. MAT is the method of choice for detecting immunoglobulin M (IgM) and immunoglobulin G class agglutinating antibodies. However, it demands great knowledge [27]. IgM ELISAs are more widely used as first screening tests because they are simpler to administer while being less specific. The assessment of serology may be problematic because the initial specimen is negative in fifty percent of cases, and the test may stay negative if antibiotics are delivered early [28]. Consequently, a negative lab test result does not rule out the diagnosis, and testing must be performed 7 to 14 days later. Molecular techniques may identify Leptospira nucleic acids in the blood days after the beginning of clinical symptoms.

Another very rare occurrence, but not as uncommon as our presented case, was of a female patient 34 years old with HBV and EBV infection. This case was reportedly the first to be documented in the medical record [29]. Epigastric discomfort and significant acute hepatitis expressed as jaundice, hyperbilirubinemia, increased transaminases, and coagulopathy were the systemic manifestations. The woman was confirmed with acute HBV coinfection with EBV, resulting in chronic hepatitis B. The presentation was severe during her acute illness, but there were no signs of hepatic encephalopathy. The patient’s transaminases and total bilirubin steadily recovered over two weeks, and she was released from the hospital. Her EBV DNA test was negative over the duration of her three-week illness. The best treatment of HBV coinfections in this instance is currently in flux. Current HBV coinfection guidelines include identifying the dominant virus and treating appropriately [30]. Due to the risk of mutual inhibition, treating one virus may enable the suppressed virus to become more active and exacerbate the course of the disease.

## 4. Conclusions

In conclusion, even though HAV and EBV infections have different transmission routes, both may be acquired simultaneously, when both incubation periods can overlap. The incubation period of HAV varies between two to six weeks, for leptospirosis, between one and four weeks, and for EBV, between four to seven weeks. If symptoms appear simultaneously, the patient may suffer from acute liver damage, which can be treated with supportive therapy in the case of HAV and EBV, and antibiotics for leptospirosis, respectively. It is also possible that the Leptospira co-infection actually can help reduce liver damage caused by HAV and EBV due to competitive inhibition. Although there are no sufficient data in the literature as a reference for this case, our patient was successfully treated for all three infections, and the evolution was favorable with early antibiotic treatment for leptospirosis. When a clinician meets a patient with elevated levels of blood transaminases and altered clinical status, it is crucially important to conduct a very thorough anamnesis because it might be more conclusive than extensive laboratory findings and a deeper understanding of the patient’s condition.

## Figures and Tables

**Table 1:** Laboratory findings during hospitalization.

Laboratory Tests	Normal Range	In the Emergency Room of the County Hospital	1 Day After Admission	5 Days After Admission	8 Days After Admission	12 Days After Admission	15 Days After Admission	20 Days After Admission
Leucocytes	4000–10,000/µL	7200/µL	7940/µL	7450/µL	6900/µL	6350/µL	6400/µL	6580/uL
Lymphocytes	25–40%	29.7%	28.8%	29.0%	29.2%	29.9%	31.1%	27.8%
Monocytes	5.5–10%	6.7%	14.4%	16.5%	17.1%	17.9%	19.0%	16.3%
Neutrophils	55–65%	63.6%	53.6%	51.8%	50.8%	50.0%	47.2%	54.8%
Prothrombin time	9.4–12.1 s	11.8 s	12.4 s	13.0 s	13.2 s	13.0 s	12.1 s	11.5 s
ALT	0–40 U/L	3911 U/L	1611 U/L	1128 U/L	661 U/L	181 U/L	298 U/L	503.8 U/L
AST	0–41 U/L	2384 U/L	503 U/L	405 U/L	263 U/L	72.6 U/L	115 U/L	246.4 U/L
Total Bilirubin	0–1.2 mg/dL	4.9 mg/dL	7.0 mg/dL	7.56 mg/dL	8.13 mg/dL	10.38 mg/dL	6.29 mg/dL	3.76 mg/dL
Direct Bilirubin	0–0.2 mg/dL	4.4 mg/dL	6.38 mg/dL	6.98 mg/dL	7.66 mg/dL	8.94 mg/dL	5.85 mg/dL	3.26 mg/dL
Cholinesterase	5320–12,920 U/L	6502 U/L	6351 U/L	6086 U/L	6641 U/L	6934 U/L	6885 U/L	6872 U/L
Creatinine	0.6–1.2 mg/dL	0.8 mg/dL	0.89 mg/dL	0.99 mg/dL	0.96 mg/dL	1.12 mg/dL	1.15 mg/dL	1.09 mg/dL
Vitamin D	20–40 ng/mL	17 ng/mL	17 ng/mL	19 ng/mL	20 ng/mL	21 ng/mL	23 ng/mL	24 ng/mL
CRP	<10 mg/L	94 mg/L	92 mg/L	58 mg/L	33 mg/L	7 mg/L	3 mg/L	1 mg/L
Procalcitonin	<0.5 ng/mL	1.2 ng/mL	1.5 ng/mL	0.73 ng/mL	0.31 ng/mL	0.28 ng/mL	0.22 ng/mL	0.2 ng/mL
Proteinuria	<150 mg/day	Negative	Negative	Negative	Negative	Negative	Negative	Negative
Hematuria	0–2 RBC	3–5 RBC	3–5 RBC	3–5 RBC	0–2 RBC	0–2 RBC	0–2 RBC	0–2 RBC
IgM anti-VCA-EBV antibodies	Negative	/	Positive	/	/	/	/	/
IgM anti-HAV antibodies	Negative	/	Positive	/	/	/	/	/
Leptospira complement fixation test	Negative	/	Positive	/	/	Negative	/	Negative

ALT—alanine aminotransferase; AST—aspartate aminotransferase; CRP—C-reactive protein; RBC—red blood cells; EBV—Epstein–Barr virus; HAV—hepatitis A virus.

## Data Availability

Not applicable.
